# Transplanting Hair

**Published:** 1873-07

**Authors:** 


TRANSPLANTING HAIR.—The successful transferring of skin and flesh to assist the recovery of wounds, has induced some one to experiment on hair, and the result is a process of removing portions of the scalp, with the hair on, from some luxuriant head, and planting it on the victim of baldness. A contemporary points out that it may soon become fashionable to wear hair of various hues and shades, thereby producing the most singular and beautiful effects of color; or the hair might be made to appear white, green, blue, or red, at the owner's option, and by various ways of disposing it. "Take, in due proportions, hair of all the prismatic tints, rumple it, and immediately you have white hair; comb it in another way and there is your purple, your ultramarine, your yellow, or any possible hue." If these directions are followed, the recognition of the original color of the head may require the use of the spectroscope.

